# Hologenomics: Systems-Level Host Biology

**DOI:** 10.1128/mSystems.00164-17

**Published:** 2018-04-10

**Authors:** Kevin R. Theis

**Affiliations:** a Department of Microbiology, Immunology and Biochemistry, Wayne State University School of Medicine, Detroit, Michigan, USA; b Perinatal Research Initiative in Maternal, Perinatal and Child Health, Wayne State University School of Medicine, Detroit, Michigan, USA

**Keywords:** animal behavior, holobiont, hologenome, microbiome, precision medicine

## Abstract

The hologenome concept of evolution is a hypothesis explaining host evolution in the context of the host microbiomes. As a hypothesis, it needs to be evaluated, especially with respect to the extent of fidelity of transgenerational coassociation of host and microbial lineages and the relative fitness consequences of repeated associations within natural holobiont populations. Behavioral ecologists are in a prime position to test these predictions because they typically focus on animal phenotypes that are quantifiable, conduct studies over multiple generations within natural animal populations, and collect metadata on genetic relatedness and relative reproductive success within these populations. Regardless of the conclusion on the hologenome concept as an evolutionary hypothesis, a hologenomic perspective has applied value as a systems-level framework for host biology, including in medicine. Specifically, it emphasizes investigating the multivarious and dynamic interactions between patient genomes and the genomes of their diverse microbiota when attempting to elucidate etiologies of complex, noninfectious diseases.

## Abstract (precis)

The hologenome concept of evolution is a hypothesis explaining host evolution in the context of the host microbiomes. As a hypothesis, it needs to be evaluated, especially with respect to the extent of fidelity of transgenerational coassociation of host and microbial lineages and the relative fitness consequences of repeated associations within natural holobiont populations.

## PERSPECTIVE

**Holobionts and hologenomes: tenable objects and roots of an evolutionary hypothesis.** All animals and plants are populated by diverse and dynamic communities of microbes, and what we perceive as the broad phenotype of an individual host is necessarily an emergent product of multivarious interactions between the host’s genome, the genomes of its numerous resident microbes, and the broader environment that they collectively inhabit. Biologists and philosophers of biology are increasingly focusing on the ecological and evolutionary dynamics of host-microbial organization, integration, and function and attempting to construct general foundational arguments that provide a vocabulary and framework for effective contemporary dialogue and research on hosts in light of their ubiquitous and substantive interactions with their microbiota (1, 2). One such argument is the hologenome concept of evolution (3). The holobiont is defined as the emergent phenotype composed of a host and its resident microbiota at a given point in time. The hologenome is defined as the genetic content of the host and its microbiota. Defined as such, holobionts and hologenomes are tenable objects; germfree animals and plants are restricted to highly controlled laboratory environments. However, the hologenome concept of evolution is also a hypothesis explaining the evolution of animals and plants in the context of the microbiome. Specifically, it suggests that selection can act on transgenerational interactions between hosts and symbiotic (i.e., resident) microbes; that these networked interactions are far more numerous, complex, and dynamic than has previously been appreciated; and that progress in elucidating the underlying etiologies of complex holobiont phenotypes can be most efficaciously achieved through integrated consideration of host, microbial, and environmental features.

There are four principal tenets of the hologenome concept (1, 3). First, all animals and plants are populated by microbial communities with which they intimately interact, and these associations are often not random but rather predictable. Notably, this predictability may reflect ecological and/or evolutionary processes, and it does not imply that hosts have coevolved with their microbiota as a whole. Host microbiota are dynamic, and their structure often varies with host ontogeny. Accordingly, at any given point in time, some microbes affect the holobiont phenotype and their populations have coevolved with the host, others affect the holobiont phenotype but have not coevolved with the host, and still others neither affect the holobiont phenotype nor have coevolved with the host (2). A challenge for us as contemporary investigators of host-microbial interactions is to elucidate which populations within the microbiota fall into each category. Doing so could elucidate subcommunities of networked symbiotic microbial populations of particular influence on holobiont phenotype and provide insight into variation in the proportion of symbiotic microbial populations affecting holobiont phenotype and coevolving with their hosts in the contexts of host lifestyle, mode of reproduction, degree of sociality, and extent of parental care and broader kin interaction.

Second, the interactions between hosts and their microbiota can have fitness consequences for the holobiont. These consequences can be positive or negative—cooperation is not assumed (1, 2). In addition, although the hologenome concept suggests that these interactions can affect fitness at the holobiont level, this does not mean that the holobiont must be the only, or even the primary, unit of selection. What the hologenome concept emphasizes is that selection can viably act on the repeated and substantive interactions between hosts and their microbiota and that the number, complexity, and degree of networked interconnectedness of these interactions have historically not been accounted for in animal and plant biology.

Third, there can be transgenerational coassociation of hosts and specific microbes. Offspring can inherit microbes from their mothers through vertical transmission via sex cells, inherit them from parents or genetic relatives through horizontal transmission during reproductive processes or social interactions, and/or acquire them from the local environment anew each generation. For fidelity of transgenerational coassociation to occur in the latter case would require highly specific host-microbial cross talk to ensure colonization by the proper symbiont as well as effective policing mechanisms by the host to ensure recurrence of the target phenotype.

Fourth, genetic variation among hologenomes can arise through changes in host genomes or the genomes of the microbiota. Genetic variation is the raw material upon which selection can act, and there is ample and labile genetic variation in host microbiomes. Notably, genetic variation among the microbiota can arise within the lifetime of the host. This can provide a potential mechanism for holobionts to adjust to rapidly changing environments and for those influential traits to potentially be passed on to offspring (4).

The hologenome concept thus suggests a reconceiving of that which constitutes an individual animal or plant. It is an assertion that animals and plants are more appropriately viewed as emergent individuals than as autonomous entities. They are holobionts, networks of host and microbial cells and genes, and these dynamic networked interactions ought to be factored into any productive consideration of their evolutionary ecology. It is likely that in the evolutionary history of each host lineage, there was never an individual that competed and reproduced independently of the influence of its microbiome. Evolution via selection is inevitable when heritable phenotypic variation results in differential reproductive success within populations. The hologenome concept of evolution is a hypothesis for the evolution of animals and plants that incorporates the microbiome. As a hypothesis, it still needs to be evaluated, and the predictions requiring concerted investigation and resolution are the extent of fidelity of transgenerational coassociation of host and microbial lineages and the relative fitness consequences of these repeated associations within natural holobiont populations. The explanatory potential of the hologenome concept is commensurate with these phenomena.

### Behavioral ecologists are in a prime position to evaluate the hologenome concept of evolution.

Behavior is the primary means that animals have for mediating their circumstances within the dynamic physical and social environments that they inhabit. As such, animals’ behavioral phenotypes are principal targets of natural and sexual selection. In behavioral ecology, the individual animal is typically perceived as being the unit of selection, and the relationships between individuals’ behavioral phenotypes, underlying genetic variation, and relative reproductive success are a frequent area of inquiry. This is important in the context of the hologenome concept because it is becoming increasingly clear that animals’ microbiomes can substantially contribute to their behavioral phenotypes, protecting them from predators, increasing their foraging efficiencies and reproductive outputs, and contributing to their chemical communication systems (5, 6). For example, among marine invertebrates, symbiotic microbes can provide counterillumination or warning coloration, each serving to stave off predation. One such invertebrate meticulously cultivates the dorsal lawn of cyanobacteria providing its warning coloration, occasionally harvesting and feeding on these symbionts. Some marine fish increase their foraging efficiencies by using bioluminescent symbionts as lures to attract prey. Among some aggregating insects and scent-marking mammals, it appears that symbiotic microbes produce the odorants that their hosts use to communicate with one another (7). Each of these behavioral phenomena provides an example wherein dynamic host-microbial interactions are potentially impacting holobiont fitness. Furthermore, while transmission of microbes from mother to offspring is likely to occur during egg-laying or live-birthing processes, animals also exhibit behaviors promoting the transmission of beneficial microbial partners to their offspring. For example, many animals engage in coprophagy, wherein adults provision offspring with microbe-laden intestinal secretions. Additionally, some wasps and beetles incorporate microbial symbionts directly into their larval brood chambers to facilitate inoculation during offspring development.

Among behavioral ecology laboratories worldwide, there are likely many behavioral phenotypes under investigation that emerge via host genome and microbiome interactions. These investigations are ideal candidates for evaluating the hologenome concept of evolution because the behavioral phenotypes chosen for study are typically readily characterized and quantifiable and the investigations are often long term, spanning multiple generations within natural animal populations, with readily available metadata on parentage and genetic relatedness, relative reproductive success, and detailed social interactions among kin. In the next half-decade, I expect that behavioral ecologists will further elucidate the mechanistic and developmental contributions of the microbiome to animals’ behavioral phenotypes and begin concertedly evaluating the extent of fidelity of transgenerational coassociation of animal and microbial lineages and calculating the relative fitness consequences of these repeated associations within natural holobiont populations. This will entail not only conducting comprehensive phylogenetic marker gene surveys to characterize variation among microbiota in behaviorally relevant animal organs but also ultimately tracking associations of specific microbial strain lineages across host animal generations using microbial source tracking, genomic fingerprinting, and/or labeling techniques. The use of complementary metagenomic surveys will be critically important, as it will address the potential issue of substantial genomic variation among microbes at the strain level and the short time scale in which this variation can arise. Additionally, behavioral ecologists will begin conducting experiments in which animal microbiomes are manipulated and/or modulated in common garden laboratory, seminatural mesocosm, and natural population experiments to evaluate the extent of determinism in animal-microbiota assembly and persistence and to assess the impact on holobiont fitness (8). These investigations will be imperative for understanding the evolutionary ecology of animal-microbe interactions in general and for evaluating the explanatory potential of the hologenome concept of evolution in particular.

### Hologenomics has applied value as a systems-level framework for host biology, including in medicine.

Although the explanatory potential of the hologenome concept as an evolutionary hypothesis needs to be evaluated, a hologenomic perspective has applied value through its emphasizing the general necessity of a systems-level framework that elucidates networked interactions between host genomes and the microbiome for complex problem solving in contemporary host biology, including in human medicine. Over the last century, mortality due to infectious disease has markedly decreased; however, mortality due to noninfectious diseases, such as cancer, obesity and diabetes, and heart and lung disease, has not declined. Although the microbiome has been implicated in each of these diseases, the underlying etiologies are complex. They are not due to the pathogenic effects of one or two microbial types but rather multivarious networked interactions between the patients’ genomes, the genomes of their diverse microbiota, and their environmental circumstances (9). That is to say, the disease phenotypes are emergent and hologenomic in nature.

Evolutionary medicine emphasizes the evolutionary history of humans in the context of disease and suggests that our rapidly changing environments have resulted in our being genetically mismatched to modernity (10). This approach would likely be more effective when viewed through a hologenomic perspective because we may be mismatched with our contemporary microbiomes as well (11). Specifically, it has been suggested that numerous feats of humankind, including harnessing the power of fire, the agricultural revolution, caesarean deliveries, the advent of antibiotics and detergents, and the widespread manufacturing of processed foods, have sequentially reduced the diversity of symbiotic microbes inhabiting our bodies. The hypothesis is that the resultant reduction in historically reliable human-microbial associations has directly contributed to the rise in chronic, noninfectious disease (11). Precision medicine is an emerging discipline in which variation in the human genome is used to predict, manage, and treat disease. Given that the majority of unique genes associated with our bodies are microbial in origin and that these genes greatly contribute to our phenotypes, precision approaches to disease are also likely to be more effective when viewed through a hologenomic lens (12, 13). Systems biology focuses on the emergent properties of complex biological challenges, and a hologenomic perspective emphasizes that solutions in host biology are facilitated by focusing on the dynamic and networked interactions of hosts and their diverse microbiota. Some critics of hologenomic perspectives suggest that investigative efforts in host-microbial evolutionary ecology are most productively directed toward identifying and validating the developmental and mechanistic interactions between specific established pathogens and/or obligate symbiotic microbes and their hosts. While such investigations have been and will continue to be highly valuable, medical microbiology studies focused on bipartite or tripartite host-microbial associations are unlikely to yield broad understanding of the emergent properties underlying complex disease phenotypes, such as cancer, obesity, diabetes, heart disease, and obstetric syndromes.

In clinical investigations of microbiota in disease, it is common to evaluate if there are predictable associations, based on patterns of a phylogenetic marker gene such as the 16S rRNA gene, between specific microbial phylotypes and incidences, or cases, of disease. However, this may be insufficient in elucidating underlying microbial etiologies of complex disease for several reasons. First, univariate analyses of the differential distribution or abundance of particular phylotypes between cases and controls may not capture the multivarious synergistic interactions among members of subcommunities contributing to the disease phenotype. Notably, these subcommunities may vary among stratified patient populations as a reflection of their own evolutionary history. Indeed, given the highly personalized nature of the microbiome, these studies will also optimally be longitudinal, with each sampled individual serving as his or her own control. Second, focusing on phylotypes does not necessarily account for strain-level variation in the metabolic or virulence potential of resident microbes, nor does it account for potential functional redundancy among microbes with respect to their capacity for eliciting disease phenotypes. Third, noninfectious polymicrobial disease is a product of host responses to microbial stimuli, so clinical investigations of microbiota independent of personalized host immune response data are unlikely to be as informative as they could otherwise be. In the next half-decade, I expect a marked increase in committed interdisciplinary collaborations between microbial ecologists, immunologists, bioinformaticists, and clinicians that facilitate rapid progress toward understanding complex, noninfectious diseases. Progress will be greatest when efforts are focused on developing integrated, longitudinal models of environmental conditions; lifestyle parameters; patient genomic variation; transcriptomic, proteomic, and metabolomic organ-specific profiles of patients and microbiota; and patient immunologic response data. Elucidating the dynamic networked interactions among these multiple levels of data will be computationally challenging; however, such hologenomic approaches will maximize the likelihood of identifying biomarkers for complex disease phenotypes. Last, the ultimate aim of clinical microbiome research is to develop sufficient understanding of the system not only to predict when host-microbiome interactions will lead to a particular disease or treatment outcome but also to effectively manage the microbiome in a personalized manner to maximize health and mitigate disease. Prebiotics and probiotics are increasingly being used to manage the microbiome: prebiotics preferentially amplify currently endogenous beneficial microbes, while probiotics supply the patient with an exogenous beneficial microbe. Prebiotics and probiotics are, in essence, targeted therapies altering the composition and/or structure of the holobiont and the hologenome (14). The emergent phenotype of the holobiont, in the context of health or disease, is being altered through targeted management of the hologenome. Notably, the response to administered prebiotics and probiotics is not uniform but rather can vary based on the original composition of the hologenome (15). Specifically, prebiotics are effective only if there is a microbe within the holobiont capable of utilizing them as a resource, and probiotics are effective only if they can effectively compete against current microbial members within the holobiont. Therefore, prebiotic and probiotic administration will be most efficaciously employed as targeted management tools in clinical medicine when used in a precision, hologenomic manner (9, 12–14).
